# Editorial: Case reports in radiation oncology: 2022

**DOI:** 10.3389/fonc.2023.1264297

**Published:** 2023-07-31

**Authors:** Ianik Plante

**Affiliations:** Human Health and Performance Contract, KBR, Houston, TX, United States

**Keywords:** radiation oncology, chemotherapy, radiotherapy, medical imaging, machine learning

This Case Reports series of Frontiers in Oncology highlights unique cases of patients that present with an unexpected diagnosis, treatment outcome, or clinical course. The cases reported are either rare cases with typical features, frequent cases with atypical features, and cases with a convincing response to new treatments.

In Laurino et al., a literature review of radio-induced sarcomas (RIS) found in patients referred to the IRCCS CROB Centro di Riferimento Oncologico della Basilicata from January 2009 to May 2022 is presented. Amongst 186 patients with histologically proven soft tissue and bone sarcomas, seven patients received a histological diagnosis of secondary RIS. This review has shown that RIS represents a possible complication for long-survivor cancer patients.

In Amidon et al., a perfusion-weighted MRI (PWI) was performed in four patients with glioblastoma to create fractional tumor burden (FTB) maps to spatially distinguish active tumors from treatment-related effects. PWI was performed prior to re-resection on those who had undergone upfront pulsed low-dose-rate radiotherapy (pLDR) concurrent with temozolomide (TMZ) and who had radiographic suspicion for tumor progression at a median of 3 months (0–5 months or 0–143 days) post-pLDR. Pathologic diagnosis revealed treatment effect (n=2), a mixture of viable tumor and treatment effect (n=1), or viable tumor (n=1). In 3 of 4 cases, FTB maps were indicative of lesion volumes being comprised predominantly of treatment effect with enhancing tumor volumes comprised of a median of 6.8% vascular tumor (6.4–16.4%). This case series provides insight into the radiographic response to upfront pLDR and TMZ and the role for FTB mapping to distinguish tumor progression from treatment effect prior to redo-surgery and within 20 weeks post-radiation.

In Wu et al., the case of a 56-year-old woman with a mass on the right breast discovered in May 2015 is reported. The treatment by modified radical mastectomy and lymph node biopsy revealed that the tumor was a metaplastic squamous cell carcinoma with axillary lymph node metastasis. The following treatment was traditional adjuvant chemotherapy and radiotherapy. A recurrence in the right chest wall was found in May 2017, so the recurring lesion was resected, then the patient was given postoperative adjuvant radiotherapy and chemotherapy. In August 2019, new pulmonary and mediastinal lymph node metastases were found on the PET/CT examination. After 4 cycles of albumin paclitaxel plus cisplatin chemotherapy combined with nivolumab immunotherapy, the patient achieved complete response, then switched to nivolumab immune maintenance therapy. At the time of writing, no obvious recurring metastasis has been observed.

In Zhou et al., the first case of choroidal melanoma complicated with a macular hole and vitreous hemorrhage after stereotactic hypofractionated radiotherapy in Japan is reported. An 83-year-old male with choroidal melanoma was treated with stereotactic hypofractionated radiotherapy in January 2021. Five months later, a full-thickness macular hole developed, followed by an acute massive vitreous hemorrhage about 2 weeks later. Following confirmation of tumor regression, the patient underwent a pars plana vitrectomy and internal limiting membrane peeling. The macular hole was closed postoperatively, and the patient’s best-corrected visual acuity improved to 20/125. There was no evidence of intraocular tumor dissemination or distant metastases during follow-up. A systematic literature search only identified 10 previous cases of choroidal melanoma with a macular hole in eight reports worldwide, mainly in females. Most patients who underwent vitrectomy for complications after tumor regression achieved a good prognosis.

In Bentahila et al., the first case of second retreatment of a spinal metastasis initially irradiated with standard radiotherapy and stereotactic body radiation therapy (SBRT), who subsequently progressed with imaging-confirmed local tumor progression at the same level, is presented. After a third course of irradiation with SBRT, a complete response was achieved. After 8 months of follow-up, the patient remains free of local recurrence. A third course of vertebral irradiation for a recurrent vertebral metastasis could be used in a selected group of patients if an adequate dose is delivered to the target while observing critical tissue tolerance limits.

In Du et al., a machine learning (ML) model based on pre-treatment multimodal magnetic resonance imaging (MRI) radiomics and clinical risk factors for brain metastasis (BM) was developed and validated. In this study, 337 BM patients were included (247, 60, and 30 in the training set, internal validation set, and external validation set, respectively). Four clinical features and 223 radiomics features were selected using least absolute shrinkage and selection operator (LASSO) and Max-Relevance and Min-Redundancy (mRMR) filters. The ML model using the selected features and the support vector machine (SVM) classifier was used to predict the treatment response of BM patients to SRS therapy. In the training set, the SVM classifier that uses a combination of clinical and radiomics features demonstrates outstanding discriminative performance given the area under curve (AUC) (AUC=0.95, 95% CI: 0.93-0.97). Moreover, this model also achieves satisfactory results in the validation sets (AUC=0.95 in the internal validation set and AUC=0.93 in the external validation set), demonstrating excellent generalizability. This ML model enables a non-invasive prediction of the treatment response of BM patients receiving stereotactic radiosurgery (SRS) therapy, which can in turn assist neurologist and radiation oncologists in the development of more precise and individualized treatment plans for BM patients.

## Author contributions

IP: Writing – original draft, Writing – review & editing.

